# Paternal age and sperm DNA fragmentation independently affect embryonic ploidy but not euploid embryo implantation potential

**DOI:** 10.3389/fendo.2026.1881113

**Published:** 2026-07-28

**Authors:** Zhiming Li, Xiufeng Lin, Xubin Zhang, Xiaowu Fang, Zhanhui Ou, Junye Huo, Wanna Ke, Jiaqi Wu, Wenjuan Yu, Lan Ma, Xiaojun Wen

**Affiliations:** Reproductive Medicine Center, Boai Hospital of Zhongshan, Zhongshan, Guangdong, China

**Keywords:** embryonic aneuploidy, paternal age, paternal factors, preimplantation genetic testing, sperm DNA fragmentation

## Abstract

**Objective:**

To systematically evaluate the associations between paternal factors (age, semen parameters, body mass index, sperm DNA fragmentation index (DFI), and leukocyte count), embryonic ploidy, and the clinical implantation potential of euploid embryos.

**Design:**

Single-center retrospective cohort study.

**Subjects:**

Couples who underwent intracytoplasmic sperm injection and simultaneous preimplantation genetic testing for aneuploidy between May 2019 and September 2024, with control for maternal confounding factors.

**Main outcome measures:**

(1) The association between paternal factors and the risk of embryonic aneuploidy, derived using generalized estimating equations, with individual embryos as the unit of analysis (2). The association between paternal factors and clinical pregnancy outcomes, with individual euploid blastocyst transfer cycles as the unit of analysis.

**Results:**

We analyzed 1,410 biopsied embryos (euploid group: 744 embryos; aneuploid group: 666 embryos). Multivariate analysis revealed that advanced paternal age (≥ 35 years) and high DFI (≥ 20%) were independent risk factors for the formation of embryonic aneuploidy, with risks increasing with higher age and DFI (e.g., age ≥ 40 years: OR = 2.033; DFI ≥ 30%: OR = 2.206). The association between paternal age and aneuploidy appeared to be associated with chromosomes 6, 18, and 22, whereas high DFI manifested as a broad genomic instability effect. Both factors were associated with a reduced incidence of X-chromosome abnormalities, which may be explained by early embryonic selective elimination. Subsequent analysis of 307 euploid embryo transfer cycles revealed that no paternal indicators were significantly associated with clinical pregnancy rates.

**Conclusion:**

Advanced paternal age and high DFI are key risk factors for embryonic aneuploidy; however, their negative effects were primarily related to chromosome composition. Once euploid embryos are obtained, these paternal factors do not have a significant independent effect on subsequent implantation potential. Our findings suggest that clinical efforts should differ across treatment stages: early treatment should prioritize active management of male factors to improve euploid embryo yield, whereas treatment during the transfer phase should prioritize optimizing maternal uterine receptivity.

## Introduction

Human embryonic aneuploidy is a major cause of implantation failure and early pregnancy loss in assisted reproduction. Preimplantation genetic testing (PGT) addresses this challenge by screening embryos for chromosomal abnormalities and selecting euploid embryos for transfer, thereby improving pregnancy rates per transfer cycle in specific populations (1, 2).

Research on embryonic ploidy has long focused on maternal factors, such as female age, ovarian reserve function, and body mass index (BMI), which were traditionally thought to determine reproductive outcomes (3–5). However, modern reproductive biology research is changing this perspective. Sperm provides half of the embryonic genome and contributes to the centrosome to initiate the first mitosis. Furthermore, the DNA carried by sperm harbors complex epigenetic information, including DNA methylation, histone modifications, and non-coding RNAs, which play a crucial role in zygotic genome reprogramming and gene expression regulation in early embryos (6–9).

The role of paternal age remains controversial. Although higher paternal age is associated with a heightened risk of miscarriage and congenital anomalies in offspring (10–13), and approximately 9.9% of all aneuploidies can be traced back to paternal sources (14), the relationship between paternal age and overall aneuploidy rates remains unclear. Some studies have suggested a positive correlation between the two factors in older fathers (> 40 to ≤ 50 years) (15, 16). However, recent analyses based on oocyte donation models, investigations into paternal-specific aneuploidies, and large-sample retrospective studies have found no significant association between paternal age and overall aneuploidy rates (17–19).

The relationship between embryo ploidy and other paternal factors, such as semen parameters, paternal BMI, and sperm DNA fragmentation index (DFI), is also debated. Severe oligoasthenoteratozoospermia or high BMI may be associated with decreased euploidy rates (20–22). However, Bonus et al. found no significant association between conventional paternal clinical factors (paternal age, BMI, and semen parameters) and the incidence of “paternal-specific” embryonic aneuploidy (18). Similarly, a large-scale analysis of clinical data revealed that neither semen parameters nor paternal BMI showed a significant clinical association with aneuploidy rate in blastocyst-stage embryos (19). Furthermore, evidence for a direct impact of leukocytospermia (sperm with leukocyte counts ≥ 1×10^6^/mL) on aneuploidy rates remains insufficient. Recent studies have shown that leukocytospermia has no significant impact on fertilization rates (23–25), embryo quality, or the clinical outcomes of assisted reproduction, and that the presence of leukocytospermia does not affect the aneuploidy rate or clinical and embryological outcomes of *in vitro* fertilization cycles (26). Regarding sperm DNA fragmentation, some studies have reported an independent association with increased aneuploidy rates (27–29), whereas others observed no association after controlling for maternal factors, suggesting that the repair potential of oocytes may partly compensate for paternal DNA damage (30, 31).

Notably, implantation failures can occur after the successful selection of euploid embryos, indicating that implantation potential is regulated by factors beyond ploidy, including paternal factors. Advanced paternal age may negatively affect pregnancy, reduce live birth rates following the transfer of euploid embryos, and increase the risk of miscarriage (32); however, relevant research remains limited.

In summary, the effect of paternal factors on preimplantation embryo development, specifically embryonic ploidy and the implantation potential of euploid embryos, remains unclear. Therefore, in this retrospective cohort study, we systematically analyzed the associations between 1) paternal indicators (age, semen parameters, BMI, DFI, and leukocyte count) and embryo chromosome composition; and 2) paternal indicators and the clinical implantation outcomes of euploid embryos. The aim of this study was to provide more comprehensive evidence for the clinical assessment and intervention of paternal factors.

## Materials and methods

### Research design and patient selection

In this single-center retrospective cohort study, the cohort included couples who underwent intracytoplasmic sperm injection and simultaneous PGT-A treatment at our reproductive medicine center from May 2019 to September 2024. To maximize the control of maternal confounding factors and accurately assess the independent effects of paternal factors, we employed the following exclusion criteria to construct a homogeneous study population: (1) confirmed uterine structural abnormalities via hysteroscopy or ultrasound sonohysterography (such as submucosal fibroids, endometrial polyps, uterine septum, or intrauterine adhesions); (2) ultrasound-diagnosed adenomyosis; (3) uncontrolled endocrine disorders (such as thyroid dysfunction or hyperprolactinemia); (4) known chromosomal karyotype abnormalities in either partner; (5) systemic diseases (such as diabetes or autoimmune diseases); (6) maternal age ≥ 38 years.

First, we investigated the effects of paternal factors on ploidy. Individual embryos were used as the unit of analysis and were categorized into euploid and aneuploid groups based on PGT-A results. All mosaic embryos were included in the aneuploid group as they are generally associated with lower implantation potential and are not routinely prioritized for transfer in clinical practice. We used the euploid group as a reference and conducted univariate and multivariate logistic regression analyses based on generalized estimating equations (GEEs) to identify paternal factors associated with the risk of embryonic aneuploidy.

Second, we investigated the effects of paternal factors on the implantation potential of euploid embryos. All PGT-A cycles involving the transfer of a single euploid blastocyst were selected from the first stage. The transfer cycle was used as the unit of analysis, with cycles divided into clinical pregnancy and implantation failure groups based on whether clinical pregnancy was achieved after transfer. By controlling for maternal confounding factors, we determined whether paternal factors were independent risk factors for implantation failure.

### Ovarian stimulation, embryo culture, and biopsy

Controlled ovarian stimulation was conducted using standardized gonadotropin-releasing hormone antagonists or agonists, with adjustments based on patient condition. Follicular growth was monitored using transvaginal ultrasonography and serum estradiol levels. When at least two dominant follicles reached an average diameter of 18 mm, recombinant human chorionic gonadotropin (Merck Serono, Geneva, Switzerland) or a gonadotropin-releasing hormone agonist was administered to trigger final follicular maturation. Oocyte retrieval was conducted after 36 h. All mature oocytes retrieved were fertilized using intracytoplasmic sperm injection technology. Fertilized eggs were assessed for pronuclei and polar bodies at 16–18 h post-fertilization to determine fertilization status. Fertilized eggs were cultured in sequential culture media (Vitrolife, Gothenburg, Sweden) in a tri-gas incubator (6% CO_2_, 5% O_2_) until the blastocyst stage. Blastocysts were graded on days 5 or 6 according to Gardner criteria. Trophectoderm biopsy was performed on blastocysts with an embryo score of 3BB or higher using a laser system (Zilos-tk^®^, Hamilton Thorne, Beverly, MA, USA). Biopsied cells (n = 4–10) were washed and transferred to sterile PCR tubes for subsequent genetic analysis, and biopsied embryos were cryopreserved for 1 h.

### PGT-A testing and assessment of embryonic ploidy

High-throughput sequencing was employed to comprehensively screen embryos for aneuploidy. First, microcellular samples obtained by biopsy were subjected to whole-genome amplification using multiple annealing and looping-based amplification cycles to obtain sufficient DNA templates. Subsequently, DNA libraries were constructed from the amplified products for sequencing with a next-generation sequencing platform (Illumina MiSeq). Sequencing data were processed using the professional analysis software ChromGo (Yikon Gene, China), which systematically evaluates the copy number variation (CNV) status of all 24 chromosomes. According to the classification standards established by our laboratory, embryo ploidy status was categorized as follows: euploid embryos (normal chromosomal copy number, CNV chimerism ratio < 30%), aneuploid embryos (abnormal chromosomal copy number, CNV chimerism ratio ≥ 70%), and chimeric embryos (CNV chimerism ratio 30–70%).

### Semen analysis

Semen parameters included sperm concentration, sperm motility, progressive motility, and sperm morphology. Semen samples were obtained through masturbation and collected on the day of oocyte retrieval. After complete liquefaction in an incubator at 37 °C, samples were analyzed in strict accordance with the standard procedures of the World Health Organization “Laboratory Manual for the Examination and Processing of Human Semen.” Specifically, a computer-assisted sperm analysis system was used to determine semen volume, sperm concentration, and total sperm count. Sperm motility was assessed using an optical microscope and categorized into rates of progressively motile, non-progressively motile, and immotile sperm, and the total motility was calculated. Sperm morphology was evaluated using rigorous Kruger standards, with slides prepared using the Diff-Quik staining method. Observations of at least 200 sperm under high-power oil immersion were used to calculate the normal morphology rate.

Other semen indicators were derived from the most recent test results prior to oocyte retrieval. Leukocyte peroxidase staining was performed to diagnose leukocytospermia as follows: 10 μl of semen was mixed with an equal volume of benzidine staining working solution and incubated at room temperature for 20 min. Next, peroxidase-positive cells (i.e., leukocytes) were counted using a hemocytometer. According to World Health Organization standards, a leukocyte concentration of ≥ 1×10^6^/mL is defined as leukocytospermia.

Sperm nuclear protein maturity was assessed using aniline blue staining to evaluate the transition of sperm nuclear protein types (i.e., the extent to which histones were replaced by protamines) as follows: prepared semen smears were fixed with 4% paraformaldehyde and stained with 5% aniline blue acetic solution for 5 min. A minimum of 200 sperm were counted under high-power microscopy, and their percentages were calculated; sperm with dark blue-stained nuclei were classified as immature.

The DFI was determined using sperm chromatin structure analysis. Aliquots of the original semen sample were washed with phosphate-buffered saline and mixed with an acidic denaturation solution to partially unwind the DNA; this was followed by the addition of acridine orange staining solution. At least 5,000 sperm were analyzed using a flow cytometer, and the DFI was calculated based on the ratio of green fluorescence (double-stranded DNA) to red fluorescence (single-stranded DNA) emitted by the sperm under a 488-nm excitation laser. DFI thresholds of 20% and 30% were selected following the work of Bungum et al., who demonstrated that DFI > 20% is associated with a reduced probability of natural conception, even when conventional semen parameters are normal, whereas DFI > 30% indicates markedly diminished fertility potential and a very low chance of achieving pregnancy (33). These clinically established thresholds were adopted for all analyses in this study.

### Statistical analysis

We used SPSS version 27 (IBM Corp., Armonk, NY, USA) for statistical analysis. In the univariate analysis, continuous data were expressed as the mean ± standard deviation, and an independent samples t-test was used for intergroup comparisons. Categorical data are presented as percentages (%), and the chi-square test was used for intergroup comparisons. Given that the data included multiple embryos from the same patient, GEEs were applied to control for clustering effects and potential confounding factors (such as individual patient differences or the common effects of assisted reproductive technology operations). Logistic regression analysis was conducted on binary outcomes (e.g., whether the embryo was euploid and whether the transfer cycle resulted in a successful pregnancy) to calculate the odds ratio (OR) and 95% confidence interval (CI) for each exposure factor to assess the impact of paternal factors on embryo ploidy status and clinical implantation outcomes. Statistical significance was set at P < 0.05.

## Results

### Baseline characteristics of participants and embryos

The baseline characteristics of study participants are shown in **Table 1**. A total of 193 and 131 oocyte retrieval cycles (744 and 666 biopsied embryos, respectively) were performed in the euploid embryo and aneuploid embryo groups. No significant differences occurred between the two groups in terms of maternal age, BMI, baseline hormone levels (FSH, E2, P, LH, and AMH), number of oocytes retrieved, or history of miscarriage (all P > 0.05). For paternal factors, the average age was significantly higher in the aneuploid group than in the euploid group (35.58 ± 5.07 years vs. 34.51 ± 4.77 years, P < 0.01), whereas BMI showed no significant difference between the two groups. Regarding semen parameters, the two groups showed no significant differences in sperm concentration, total motility, progressive motility, positive peroxidase leukocyte rate, sperm nuclear immaturity rate, or abnormal sperm index. However, the sperm DFI was significantly higher in the aneuploid group than in the euploid group (23.54 ± 17.03% vs. 20.86 ± 17.85%, P = 0.04).

**Table 1:** Baseline characteristics of patients in the Chromosome aneuploid group and the Chromosome euploidy group.

Item	Chromosome euploidy group	Chromosome aneuploid group	p-value
Number of oocyte retrieval cycle, n	193	131	–
Number of biopsied embryos, n	744	666	–
Female factors			
Female age (Mean ± SD)	32.23±4.07	32.61±4.15	0.08
Female BMI (Mean ± SD)	22.06±2.66	22.24±2.69	0.217
Basal sexual hormone			
FSH (IU/L, average ± SD)	7.10±2.31	7.27±2.70	0.163
E2 (pg/mL, average ± SD)	33.33±12.14	34.21±14.31	0.213
P (ng/mL, average ± SD)	0.78±2.73	0.97±2.87	0.220
LH (IU/L, average ± SD)	5.07±3.40	4.98±2.52	0.613
AMH (ng/mL, average ± SD)	4.27±2.83	4.01±2.24	0.055
Retrieved Oocytes, (Mean ± SD)	15.55±7.78	15.18±7.47	0.371
No. of miscarriages (average ± SD)	1.75±1.40	1.82±1.43	0.307
Male factors			
Male age (Mean ± SD)	34.51±4.77	35.58±5.07	<0.01
Male BMI (Mean ± SD)	24.19±3.33	24.19±3.29	0.995
Fresh semen parameters			
Sperm concentration,(×10^6^/mL,Mean ± SD)	51.59±29.91	53.89±30.69	0.155
Sperm vitality, (%,Mean ± SD)	50.00±19.32	50.66±19.23	0.516
Progressive motility,(%,Mean ± SD)	33.70±15.61	35.98±15.52	0.745
Leukocyte peroxidase test			
<1×10^6^,n(%)	380(51.08%)	338(50.75%)	0.903
≥1×10^6^,n(%)	364(48.92%)	328(49.25%)	
Sperm nuclear immaturity,(%,Mean ± SD)	29.29±9.53	29.11±10.11	0.749
Teratozoospermia index, (%,Mean ± SD)	1.47±0.08	1.47±0.08	0.540
Sperm DNA fragmentation index,(%,Mean ± SD)	20.86±17.85	23.54±17.03	0.04

### Association between paternal factors and risk of embryonic aneuploidy

We used GEEs and logistic regression to analyze the relationship between paternal factors and the risk of embryonic aneuploidy (**Table 2**). After adjusting for maternal factors (age, BMI, AMH level, number of oocytes retrieved, and history of miscarriage), paternal age was revealed as a significant risk factor for aneuploidy. Compared to males aged < 35 years, males aged 35–40 years had a significantly higher risk of aneuploidy (OR = 1.506, 95% CI 1.058–2.143, P = 0.023), whereas males aged ≥ 40 years had the highest risk (OR = 2.033, 95% CI 1.293–3.196, P = 0.002). The DFI also demonstrated a dose-dependent relationship with aneuploidy risk, as shown by a significantly higher aneuploidy risk at DFI 20–30% than at DFI < 20% (OR = 1.593, 95% CI 1.120–2.265, P = 0.010), and the highest risk at DFI ≥ 30% (OR = 2.206, 95% CI 1.528–3.183, P < 0.001). In contrast, paternal BMI, sperm concentration, sperm motility (total and progressive motility), normal sperm morphology rate, leukocyte peroxidase positivity rate, sperm nuclear immaturity rate, and abnormal sperm index were not significantly correlated with the risk of embryonic aneuploidy.

**Table 2:** GEE and logistics analysis prediction of male-related risk factors in chromosomal aneuploidy embryos.

Item	Chromosome euploidy group vs. chromosome aneuploidy group		
	P-value	OR	95% CI
Male age (years)			
< 35	Reference	–	–
≥35 to<40	0.023	1.506	1.058-2.143
≥40	0.002	2.033	1.293-3.196
Male BMI	0.835	0.996	0.959-1.034
Sperm concentration (×10^6^/mL)			
<5	0.966	1.015	0.507-2.032
≥5 to <15	0.714	0.877	0.435-1.768
≥15	Reference	–	–
Sperm vitality			
≥40%	Reference	–	–
<40%	0.055	0.696	0.480-1.008
Progressive motility			
<32%	0.549	0.912	0.674-1.233
≥32%	Reference	–	–
Normal morphology			
<4%	0.289	0.789	0.509-1.223
≥4%	Reference	–	–
Leukocyte peroxidase test			
<1×10^6^	Reference	–	–
≥1×10^6^	0.822	0.973	0.769-1.233
Sperm nuclear immaturity			
≤30	Reference	–	–
>30	0.753	1.046	0.791-1.384
Teratozoospermia index			
<1.6	Reference	–	–
≥1.6	0.859	0.958	0.599-1.533
Sperm DNA fragmentation index			
<20%	Reference	–	–
≥20% to <30%	0.010	1.593	1.120-2.265
≥30%	<0.001	2.206	1.528-3.183

The OR (95% CI) and P values for the association between paternal factors and embryonic aneuploidy were adjusted for the following confounders: maternal age, BMI, AMH, number of oocytes retrieved, and history of miscarriage. GEE were applied to account for clustering effects among embryos from the same patient. OR: odds ratio; CI: confidence interval.

### Baseline characteristics of participants in pregnancy and non-pregnancy groups after euploid embryo transfer

The baseline characteristics of patients in the clinical pregnancy and non-pregnancy groups following euploid embryo transfer are shown in **Table 3**. A total of 307 frozen-thawed embryo transfer cycles were analyzed, including 198 cycles in the pregnancy group and 109 cycles in the non-pregnancy group. The two groups showed no significant differences in maternal factors, such as age and endometrial thickness, on the day of transfer. Additionally, no significant differences occurred between the two groups in terms of paternal age, BMI, or semen parameters (including sperm concentration, motility, progressive motility, leukocyte peroxidase positivity rate, immature sperm nucleus rate, teratospermia index, and DFI) (P > 0.05).

**Table 3:** Baseline characteristics of patients in the euploid embryo transfer clinical pregnancy group and the euploid embryo transfer clinical non-pregnancy group.

Item	euploid embryo transfer clinical pregnancy group	euploid embryo transfer clinical non-pregnancy group	p-value
FET cycles, n	198	109	–
Female factors			
Female age (Mean ± SD)	33.13±4.21	33.42±4.01	0.550
Thickness of endometrium (mm, average ± SD)	9.83±1.81	9.48±4.97	0.369
Male factors			
Male age (Mean ± SD)	35.06±4.66	35.61±5.03	0.342
Male BMI (Mean ± SD)	24.15±3.17	24.45±4.02	0.473
Fresh semen parameters			
Sperm concentration,(×10^6^/mL,Mean ± SD)	52.25±31.63	55.68±31.27	0.362
Sperm vitality, (%,Mean ± SD)	50.84±19.12	53.87±20.48	0.196
Progressive motility,(%,Mean ± SD)	33.79±14.86	35.75±18.02	0.305
Leukocyte peroxidase test			
<1×10^6^,n(%)	101 (51.01%)	51 (46.79%)	0.479
≥1×10^6^,n(%)	97 (48.99%)	58 (53.21%)	
Sperm nuclear immaturity,(%,Mean ± SD)	29.29±9.53	29.11±10.11	0.749
Teratozoospermia index, (%,Mean ± SD)	1.48±0.08	1.46±0.09	0.078
Sperm DNA fragmentation index,(%,Mean ± SD)	21.60±18.96	19.46±11.80	0.286

### Association between paternal factors and euploid embryo implantation potential

We used GEEs to conduct a multifactorial analysis of paternal factors between pregnant and non-pregnant groups (**Table 4**). After adjusting for confounding maternal factors (age, BMI, AMH, number of oocytes retrieved, number of miscarriages, and endometrial thickness), neither paternal age, male BMI, or any semen parameter (sperm concentration, sperm motility, sperm progressive motility, leukocyte peroxidase, sperm nuclear immaturity, abnormal sperm index, and sperm DFI) were significantly associated with clinical pregnancy outcomes following euploid embryo transfer. Specifically, the implantation success rate of euploid embryos for males aged ≥ 40 years was not significantly different from that for males aged < 35 years (OR = 1.183, 95% CI 0.439–3.191, P = 0.739). Similarly, a high DFI (≥ 30%) did not significantly affect the clinical pregnancy rate of euploid embryos (OR = 1.185, 95% CI 0.447–3.140, P = 0.733).

**Table 4:** GEE and logistics analysis prediction of male-related risk factors in the euploid embryo transfer clinical pregnancy group and the euploid embryo transfer clinical non-pregnancy group.

Item	Euploid embryo transfer clinical pregnancy group vs. Euploid embryo transfer clinical non-pregnancy group		
	P-value	OR	95% CI
Male age (years)			
< 35	Reference	–	–
≥35 to<40	0.821	0.916	0.426-1.968
≥40	0.739	1.183	0.439-3.191
Male BMI	0.487	0.963	0.867-1.070
Sperm concentration (×10^6^/mL)			
<5	0.784	0.757	0.103-5.556
≥5 to <15	0.142	0.199	0.023-1.714
≥15	Reference	–	–
Sperm vitality			
≥40%	Reference	–	–
<40%	0.680	1.223	0.470-3.183
Progressive motility			
<32%	0.682	0.869	0.443-1.703
≥32%	Reference	–	–
Normal morphology			
<4%	0.424	0.634	0.208-1.936
≥4%	Reference	–	–
Leukocyte peroxidase test			
<1×10^6^	Reference	–	–
≥1×10^6^	0.397	1.250	0.746-2.096
Sperm nuclear immaturity			
≤30	Reference	–	–
>30	0.577	0.837	0.447-1.565
Teratozoospermia index			
<1.6	Reference	–	–
≥1.6	0.283	0.547	0.182-1.646
Sperm DNA fragmentation index			
<20%	Reference	–	–
≥20% to <30%	0.789	0.899	0.413-1.959
≥30%	0.733	1.185	0.447-3.140

The OR (95% CI) and P values for the association between paternal factors and embryonic aneuploidy were adjusted for the following confounders: maternal age, BMI, AMH, number of oocytes retrieved, history of miscarriage, and endometrial thickness. GEE were applied to account for clustering effects among embryos from the same patient.

### Specific impact of paternal age on the risk of embryonic aneuploidy

To investigate the specific impact of paternal age on the risk of embryonic aneuploidy, we analyzed the incidence of chromosomal abnormalities across different age groups. Paternal age had a pronounced effect on the type of embryonic chromosomal abnormality (**Table 5**). Among the autosomes, the distribution of abnormalities in chromosomes 6, 18, and 22 differed significantly among the three age groups (P = 0.003, 0.038, and 0.003, respectively). Specifically, the incidence of chromosome 6 abnormalities was highest in the 35–40 age group (6.94%), which was significantly higher than that in the < 35 (3.46%) and ≥ 40 (1.78%) age groups. In contrast, the abnormality rates for chromosomes 18 and 22 showed an increasing trend with increasing paternal age, peaking in the ≥ 40 age group (7.56% and 12.00%, respectively).

**Table 5:** The frequency of each chromosome aneuploidy impact on the embryo by paternal age.

Chromosome	The frequency of abnormality occurrence for each chromosome			
	<35	≥35 to <40	≥40	P-value
1	27	23	5	0.111
2	19	18	14	0.346
3	16	17	6	0.783
4	17	27	12	0.286
5	22	15	6	0.311
6	16	32	4	0.003
7	21	18	8	0.803
8	21	14	6	0.338
9	18	21	3	0.101
10	19	13	7	0.541
11	18	16	8	0.941
12	18	14	6	0.643
13	19	19	6	0.598
14	12	19	8	0.458
15	17	27	12	0.286
16	47	33	18	0.253
17	12	8	6	0.615
18	16	18	17	0.038
19	15	16	13	0.233
20	15	7	8	0.159
21	21	26	19	0.117
22	22	37	27	0.003
X	34	19	5	0.008
Y	1	4	1	0.371

The distribution of X-chromosome abnormalities was also significantly related to paternal age (P = 0.008) but exhibited the opposite trend, with the highest incidence in males < 35 years of age (7.34%) and the lowest incidence in males aged ≥ 40 years (2.22%). The incidence of abnormalities in other chromosomes, including autosomes 1–5, 7–17, 19–21, and the Y chromosome, did not differ significantly among the three groups (P > 0.05). It should be noted that these results are exploratory and should be validated through further research.

### Specific impact of DFI on the risk of embryonic aneuploidy

Among all types of chromosomal abnormality (including chromosomes 1–22 and the Y chromosome), only abnormalities in the X chromosome showed significantly different incidence rates across the three DFI groups (P = 0.039; **Table 6**). Specifically, the rate of X-chromosome abnormalities was highest in the DFI<20% group (8.62%), followed by the DFI ≥ 30% group (4.87%), and lowest in the DFI 20–30% group (3.50%). Notably, although the incidence of chromosome 21 abnormalities was not significantly different across the three DFI groups (P = 0.081), the abnormality rate increased with increasing DFI.

**Table 6:** The frequency of each chromosome aneuploidy impact on the embryo by DFI.

Chromosome	The frequency of abnormality occurrence for each chromosome			
	<20	≥20% to <30%	≥30%	P-value
1	32	13	10	0.237
2	23	9	19	0.316
3	23	6	10	0.707
4	27	14	15	0.532
5	26	6	11	0.535
6	23	10	19	0.337
7	29	10	8	0.228
8	23	10	8	0.415
9	25	6	11	0.609
10	22	4	13	0.330
11	22	8	12	0.996
12	19	5	14	0.383
13	24	6	14	0.600
14	27	5	7	0.111
15	28	13	15	0.744
16	50	20	28	0.930
17	16	4	6	0.661
18	26	9	16	0.851
19	19	10	15	0.430
20	12	8	10	0.343
21	23	12	23	0.081
22	40	22	24	0.271
X	40	7	11	0.039
Y	6	4	2	0.403

## Discussion

In this study, we systematically examined the associations between paternal factors and the preimplantation development of embryos, characterized by stage specificity and heterogeneity, through a rigorous retrospective cohort analysis, with control of maternal confounding factors. Our results suggest that advanced paternal age (≥ 35 years) and a high DFI (≥ 20%) are independent risk factors for embryonic aneuploidy. However, once embryos achieved euploid status, as indicated by chromosomal screening, paternal indicators (age, BMI, DFI, semen parameters, leukocytes, and nucleoproteins) had no significant independent effects on clinical implantation potential. Further analysis indicated that advanced paternal age specifically affected certain autosomes (chromosomes 6, 18, and 22), whereas a high DFI presented a widespread genomic instability effect. Notably, both factors were associated with a reduced incidence of X-chromosomal abnormalities. These results imply that the negative effects of paternal factors are concentrated and terminated at the embryonic chromosomal composition stage; once this ‘quality checkpoint’ is surpassed, the success of embryo implantation is predominantly governed by maternal factors, such as uterine receptivity.

The effect of paternal age on embryonic ploidy has long been debated. Previous studies have yielded inconsistent conclusions, leading to ambiguity regarding the importance of paternal age during clinical consultations. Some rigorously designed studies utilizing oocyte donation models to eliminate maternal age as a confounding factor found no significant association between paternal age and aneuploidy rate (17). Subsequent research using large sample sizes and rigorous study designs also found that paternal age had no significant clinical relevance on overall aneuploidy rates (19). However, recent studies have presented contradictory evidence, suggesting that paternal age significantly influences embryonic euploidy (16) and may be associated with an increased risk of specific types of abnormalities, such as segmental aneuploidy, particularly in males aged > 50 years (34). After controlling for maternal confounding factors, we confirmed that paternal age is an independent risk factor for embryonic aneuploidy, with a dose-dependent increase in risk. Mechanistically, advanced paternal age is linked to DNA fragmentation, oxidative stress, chromatin remodeling, and sperm epigenetic alterations that may influence early embryo development and genome stability (35). These pathways provide a plausible biological explanation for the observed associations between paternal age, elevated DFI, and increased embryonic aneuploidy risk.

Notably, we present the first chromosome-specific map of the effects of paternal age within the same cohort, which were not uniformly distributed but significantly enriched in chromosomes 6, 18, and 22. Notably, the rate of chromosome-22 abnormalities in the ≥ 40 age group was 2.5 times higher than that in younger age groups. These results provide crucial evidence that the impact of paternal age may be chromosome specific. Accordingly, research focusing on the overall aneuploidy rate without analyzing changes at the chromosomal level may dilute the effects of paternal factors owing to the large number of unaffected chromosomes. This finding shifts the research focus from generalized risk assessment to an exploration of the underlying biological mechanisms, including whether the stability of specific chromosome centromeres and mechanisms of meiotic checkpoints exhibit specific declines with advancing paternal age (36–38). The preferential involvement of specific chromosomes in aneuploidy is not without precedent. Chromosomes 18 and 22 are consistently reported as frequent chromosomes in aneuploid conceptions (39). Moreover, limited fluorescence *in situ* hybridization studies on sperm have provided direct evidence for preferential paternal age effects on specific autosomes. Specifically, Martin et al. demonstrated that paternal age significantly increased the frequency of chromosome-1 disomy but not that of chromosome-12 disomy in human sperm (40), and Robbins et al. reported that paternal age significantly increased the frequency of sex chromosome disomy but had no effect on chromosome-8 disomy (41). Although these findings imply that paternal age has chromosome-specific effects on aneuploidy, and the observed enrichment of chromosomes 18 and 22 is biologically plausible given their high baseline susceptibility to segregation errors, the specific contribution of paternal age to these autosomal abnormalities requires additional verification through further dedicated studies.

The complete sperm DNA structure is required for the accurate transmission of paternal genetic information during fertilization. Therefore, breakage of sperm DNA strands is a major cause of genetic material damage in male gametes, and a high DFI is associated with low fertilization rates (42–44), poor embryo quality, and high miscarriage rates; however, a direct association between DFI and embryonic aneuploidy has not previously been confirmed (45–47). Although some studies have suggested an association between high DFI and an increased incidence of segmental and paternal whole-chromosome aneuploidies (21, 27–29), other studies failed to confirm this association, even after controlling for maternal factors (30, 31). This inconsistency may stem from differences in detection methods, threshold settings, and degree of control over confounding factors. Our GEE-based multifactorial analysis confirmed that a high DFI (≥ 20%) is a significant independent risk factor for embryonic aneuploidy, with a clear dose–response relationship (OR = 1.593 for DFI 20–30%; OR = 2.206 for DFI ≥ 30%). Furthermore, unlike the chromosome-specific effects of paternal age, a high DFI is not significantly associated with an increased incidence of specific autosomal abnormalities. This raises the possibility that high DNA fragmentation may not directly induce errors in the separation of specific chromosomes but rather induces widespread genomic instability. The underlying mechanism may be related to oxidative stress, as reactive oxygen species cause extensive damage to sperm DNA. When damaged sperm participate in fertilization, the compromised paternal genome interferes with the fidelity of DNA repair and replication during first mitosis of the zygote, uniformly increasing the probability of errors across all chromosomes, resulting in a general increase in the risk of embryonic aneuploidy (48–50).

Notably, we observed a unique response pattern for the X chromosome, which showed reduced incidence with advanced paternal age (≥ 40 years) and high DFI (≥ 20%). Although the rate of X-chromosome abnormalities for DFI ≥ 30% was lower than that for DFI 20–30%, it remained significantly lower than that for DFI < 20%. This phenomenon contradicts the behavioral patterns observed in autosomes and may be explained by a selective elimination mechanism targeting paternal X-chromosome defects, primarily driven by the extreme haplo-insensitivity of the X chromosome (51). When advanced paternal age or high DFI significantly increase the risk of severe abnormalities (such as 45,X monosomy), embryos carrying lethal defects could experience developmental arrest at early stages (such as the cleavage stage) because of their inability to establish the gene dosage balance necessary for continued development before blastocyst formation (52). Therefore, embryos that survive to the blastocyst biopsy stage would represent a screened group of ‘survivors,’ contributing to a reduced detection rate of X chromosome abnormalities. This phenomenon of ‘survivor bias’ raises the possibility that the harm posed by paternal factors exists at an earlier stage of embryonic loss prior to the PGT-A detection period; however, this hypothesis remains speculative and requires validation. Notably, embryos with paternal X-chromosome deletions could face even more severe survival challenges than those with maternal deletions (53, 54), which may further amplify the impact of paternal factors on this selection process.

The effect of paternal factors on the implantation potential of euploid embryos remains a critical yet understudied topic in the field of assisted reproduction. Kaarouch et al. found that a high DFI was associated with significantly lower implantation and pregnancy rates for embryos diagnosed as euploid (55), which differs from our results. We believe that this discrepancy may be related to the different developmental stages at which embryo biopsies for genetic assessment are performed, which are based on trophoblast biopsies and comprehensive chromosomal screening of blastocysts on the fifth or sixth day. We observed that once embryonic chromosomes were confirmed as euploid, paternal factors (including age, BMI, semen parameters, DFI, leukocytosis, and sperm nuclear proteins) were no longer independently associated with subsequent clinical implantation outcomes. This suggests that the detrimental effects of paternal factors may primarily occur during the early stages of embryonic development (such as chromosomal replication and early cleavage), affecting the rates of blastocyst formation and aneuploidy. However, once embryos successfully overcome these early obstacles, their subsequent implantation potential may be primarily determined by maternal factors, such as maternal uterine receptivity.

The primary strength of this study lies in its methodological rigor. First, by establishing strict inclusion and exclusion criteria (such as limiting female participant age to < 38 years and excluding major uterine pathological factors), we constructed a highly homogeneous patient cohort that effectively controlled for maternal confounding factors. Second, we employed GEEs to correct for clustering effects among multiple embryos from the same patient, thereby enhancing the efficiency and accuracy of statistical tests. Notably, we deconstructed the impact of paternal factors into two independent phases: embryonic ploidy formation and euploid embryo implantation potential. This allowed us to delineate the boundaries of the influence of different paternal factors.

However, this study also had several limitations. First, owing to the single-center retrospective design, the results may have limited generalizability to different populations; all observed associations should be interpreted as correlational rather than causal, and the proposed underlying mechanisms require prospective or functional validation. Second, the genetic analysis was based on blastocyst-stage biopsies, which cannot capture abnormal embryos that experience developmental arrest before blastocyst formation; thus, the impact of paternal factors on early embryonic loss may be underestimated. Third, we did not include more advanced indicators of sperm function and cell biology, such as reactive oxygen species levels, sperm chromatin structure, and centrosome function. Fourth, although our results revealed an epidemiological association between both advanced paternal age and a high DFI and embryo ploidy, the underlying molecular mechanisms were not elucidated. Thus, further basic research is required to determine whether this association interferes with the epigenetic reprogramming of specific chromosomes, accelerates telomere loss, or affects the fidelity of zygotic mitosis. Fifth, oocyte age remains the main determinant of embryo aneuploidy, with subtle differences in oocyte quality potentially influencing outcomes; however, we did not directly measure oocyte quality beyond conventional parameters; for example, by determining meiotic spindle integrity and mitochondrial function. Thus, although we adjusted for maternal age and AMH in the GEEs, residual confounding by maternal oocyte quality cannot be entirely excluded. Finally, implantation outcomes may be influenced by endometrial receptivity and embryo–endometrial synchrony, which were not directly assessed. Although endometrial thickness was measured, molecular markers of receptivity were unavailable; thus, their potential influence cannot be excluded.

In summary, we provide evidence for the association between paternal factors and assisted reproductive outcomes. Advanced paternal age and high sperm DNA fragmentation were both identified as significant risk factors for embryonic aneuploidy; however, these factors may exert their effects through different mechanisms. We propose that the impact of advanced paternal age is chromosome-specific, whereas DNA fragmentation contributes to broader genomic instability. Both factors primarily reduce the probability of pregnancy by disrupting the normal composition of embryonic chromosomes, but have a relatively minor effect on the implantation potential of selected euploid embryos. Based on our findings, we suggest prioritizing clinical efforts according to the treatment stage. Specifically, for couples with advanced paternal age or high DFI, early management could focus on modifiable male factors to improve euploid embryo yield, whereas optimizing maternal uterine receptivity may become more relevant during the transfer phase.

## Data Availability

The original contributions presented in the study are included in the article/supplementary material. Further inquiries can be directed to the corresponding author.
